# Restarting Neglected Tropical Diseases Programs in West Africa during the COVID-19 Pandemic: Lessons Learned and Best Practices

**DOI:** 10.4269/ajtmh.21-0408

**Published:** 2021-10-20

**Authors:** Achille Kabore, Stephanie L. Palmer, Ernest Mensah, Virginie Ettiegne-Traore, Rose Monteil, Franck Sintondji, Justin Tine, Daniel Tesfaye, Kisito Ogoussan, Diana Stukel, Brian B. Fuller, Katherine Sanchez, Bolivar Pou, Benoit Dembele, Angela Weaver, Steven Reid, Marie Denise Milord, Yao Kassankogno, Anders Seim, Joseph Shott

**Affiliations:** ^1^ FHI 360, Washington, District of Columbia;; ^2^ FHI 360, Accra, Ghana;; ^3^ FHI 360, Abidjan, Cote d’Ivoire;; ^4^ FHI 360, Dakar, Senegal;; ^5^ FHI 360, Cotonou, Benin;; ^6^ Helen Keller International, New York, New York;; ^7^ Helen Keller International, Dakar, Senegal;; ^8^ Health and Development International, Newburyport, Massachusetts;; ^9^ Health and Development International, Lomé, Togo;; ^10^ Health and Development International, Fjellstrand, Norway;; ^11^ Neglected Tropical Diseases Division, Office of Infectious Diseases, Global Health Bureau, USAID, Washington, District of Columbia

## Abstract

Countries across West Africa began reporting COVID-19 cases in February 2020. By March, the pandemic began disrupting activities to control and eliminate neglected tropical diseases (NTDs) as health ministries ramped up COVID-19–related policies and prevention measures. This was followed by interim guidance from the WHO in April 2020 to temporarily pause mass drug administration (MDA) and community-based surveys for NTDs. While the pandemic was quickly evolving worldwide, in most of West Africa, governments and health ministries took quick action to implement mitigation measures to slow the spread. The U.S. Agency for International Development’s (USAID) Act to End NTDs | West program (Act | West) began liaising with national NTD programs in April 2020 to pave a path toward the eventual resumption of activities. This process consisted of first collecting and analyzing COVID-19 epidemiological data, policies, and standard operating procedures across the program’s 11 countries. The program then developed an NTD activity restart matrix that compiled essential considerations to restart activities. By December 2020, all 11 countries in Act | West safely restarted MDA and certain surveys to monitor NTD prevalence or intervention impact. Preliminary results show satisfactory MDA program coverage, meaning that enough people are taking the medicine to keep countries on track toward achieving their NTD disease control and elimination goals, and community perceptions have remained positive. The purpose of this article is to share the lessons and best practices that have emerged from the adoption of strategies to limit the spread of the novel coronavirus during MDA and other program activities.

## INTRODUCTION

Neglected tropical diseases (NTDs) are a diverse group of 20 conditions found in tropical and subtropical settings across 149 countries. They affect more than one billion people and cost developing economies billions of dollars every year.^1^^,^^2^ In Africa alone, NTDs cause an estimated 200,000 deaths per year in the absence of appropriate treatment.^3^^,^^4^ Populations living in poverty, without sufficient access to clean water and adequate sanitation infrastructure, and who live in close contact with infectious vectors and domestic animals and livestock are the most affected.

Some NTDs can be controlled or eliminated with the assistance of mass drug administration (MDA), sometimes also called preventive chemotherapy, a strategy in which NTD medicines are provided to at-risk persons living in an endemic geographic area. According to the WHO, MDA is a cost-effective approach that, over time, can lead to national control or elimination of PC-NTDs such as lymphatic filariasis, onchocerciasis, trachoma, schistosomiasis, and soil-transmitted helminthiases.^5^

The U.S. Agency for International Development (USAID) funds the Act to End NTDs | West Program (Act | West), led by FHI 360, to support countries to control and/or eliminate these five NTDs. In addition to the lead organization Family Health International 360 (FHI 360), the Act | West consortium includes five partners: Helen Keller International (Helen Keller), Health and Development International (HDI), World Vision, the AIM Initiative, and Deloitte. The program operates in 11 countries: Benin, Burkina Faso, Cameroon, Côte d’Ivoire, Ghana, Guinea, Mali, Niger, Senegal, Sierra Leone, and Togo. The main activities of the Act | West program are to support national NTD programs to implement MDA and surveys to monitor the effectiveness and impact of MDA, as well as surveys for post-MDA surveillance, such as transmission assessment surveys (TAS) for lymphatic filariasis, stop MDA surveys for onchocerciasis, and trachoma impact and surveillance surveys.

With the global emergence of the COVID-19 pandemic in 2020, national program managers, partners, donors, and the WHO became concerned that activities, such as MDA and field-based surveys, could contribute to the spread of severe acute respiratory syndrome coronavirus 2 (SARS-CoV-2) infections, given their large scale and mass convenings. In the African region, by May 2020, COVID-19 cases were reported in all NTD-endemic countries. By the end of November 2020, approximately 1.48 million cases and 24,000 deaths had been reported.^3^ As infections spread, pandemic response–related restrictions caused disruption to essential NTD activities.

On April 1, 2020, the WHO issued interim guidance recommending that “community-based surveys, active case-finding activities and mass treatment campaigns for neglected tropical diseases be postponed until further notice.”^6^ In May 2020, the WHO issued additional guidance that reaffirmed this recommendation but noted that “countries should monitor and re-evaluate at regular intervals the necessity for delaying these activities.”^7^ In July 2020, the WHO issued guidance for countries as they began to consider restarting NTD campaigns and surveys. Primarily, countries were asked to consider the risks and benefits of resuming NTD activities and to take measures to decrease the risk of SARS-CoV-2 transmission associated with NTD activities.^8^

## MATERIALS AND METHODS

**Figure 1.**
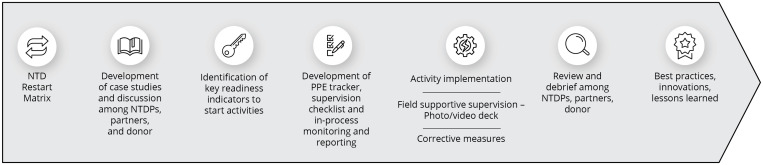
The steps and process of resuming neglected tropical disease (NTD) activities. NTDP = NTD program; PPE = personal protective equipment.

In line with the WHO recommendations and guidance issued in April 2020, USAID advised Act | West to temporarily suspend NTD field activities. A temporary suspension of activities provided time for national programs, with the support of Act | West implementing partners, to develop risk assessment tools and processes, standard operating procedures to limit SARS-CoV-2 transmission during activities, and weigh the risks and benefits of restarting activities (Figure 1).

**Figure 2.**
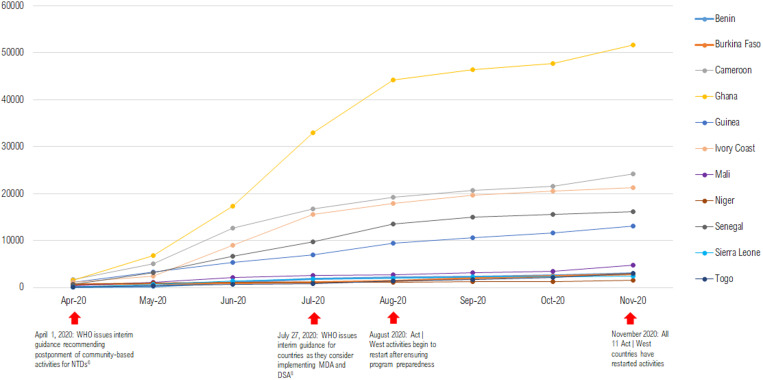
Cumulative number of COVID-19 cases by country, April–November 2020. DSA = disease-specific assessment; MDA = mass drug administration; NTD = neglected tropical disease. Source: OurWorldInData.org. Available at: https://ourworldindata.org/coronavirus.

As the pandemic continued, national programs, donors, and implementing partners grew concerned that the postponement of NTD activities would threaten or erode program gains toward control or elimination.^9^ However, the apparent success of mitigation measures put into place by ministries of health in the 11 countries enabled strict lockdowns to be lifted after a few weeks, providing opportunities for countries to restart planned NTD activities. Under Act | West, the first step in the process to restart activities was the development of an “NTD activity restart matrix” and case studies.

## NTD ACTIVITY RESTART MATRIX

The Act | West NTD activity restart matrix for the COVID-19 context compiled considerations essential for the restart of activities, including the existence and status of mitigation measures, risk communication and community engagement strategies, both of which have helped build confidence to resume activities (Supplemental Material 1). The matrix was not a decision-making tool per se; instead, it was used to invite discussion on challenges and opportunities to restart, along with any additional support needed to limit spread of SARS-CoV-2 and ensure successful program implementation. This matrix of considerations for restarting NTD programs has five domains:

1. COVID-19 epidemiology at the national and subnational levels: Act | West country teams monitored community transmission risk by reviewing data from weekly “COVID-19 Situation Reports” compiled at the national level and often containing district-level information, enabling national programs and Act | West to monitor the situation in the districts where activities were scheduled to take place. Figure 2 shows cumulative number of COVID-19 cases over time by country.
2. Risk communication and community engagement: national programs, in collaboration with Act | West, investigated community knowledge and perceptions of the pandemic through community dialogue with community drug distributors (and community leaders. National NTD programs developed communication strategies to disseminate clear messages about SARS-CoV-2 prevention and NTD programming. Careful consideration was taken to ensure adequate social mobilization in advance.
3. Policies: Act | West, in coordination with national programs and USAID, reviewed policies issued by ministries of health and their COVID-19 task forces, as well as the WHO, to understand whether conducting NTD control activities would be possible and what modifications would be required.
4. Country capacity and readiness for NTD program restart: in coordination with national programs, Act | West assessed the availability of NTD drugs, diagnostic tools, personal protective equipment, financial and human resources, and appropriately sized and ventilated training venues).
5. Taking stock of and learning from other public health campaigns: Although most countries initially postponed public health interventions, mass campaigns for malaria seasonal chemoprophylaxis and bed-net distribution, as well as vitamin A distribution, were still conducted as planned in Mali, Senegal, and Côte d’Ivoire because they were designated critical lifesaving measures. The national programs carefully reviewed the documentation produced from these mass campaigns, including photos, videos, and reports, to learn about the challenges of conducting mass campaigns during the pandemic.

The matrix was used to guide subsequent discussions with national NTD programs and other government entities involved in NTD activities (e.g., departments of education, social affairs) about potential implementation challenges and approaches to consider for safe restart of NTD activities. In a few countries (Cameroon, Guinea, and Côte d’Ivoire), different partners were working with the national programs to restart activities during the same timeframe by assessing risks and readiness. In these cases, the specific tools may have differed between partners and donors; however, the tools encompassed similar factors and considerations.^10^

## CASE STUDIES

After the completion of the NTD activity restart matrix, the information was used to develop “case studies,” which pulled salient points from each matrix into a brief PowerPoint to facilitate discussions between the national programs, Act | West team and USAID.

In several countries, the case studies were also informed by the experiences of NTD program managers who had participated in national COVID-19 task forces and collaborated on developing and rolling out mitigation measures. Their involvement with these task forces facilitated the smooth continuance of NTD activities, including assessment of capacity to handle potential COVID-19 cases, as part of the preparation for resuming field activities in some countries. In Côte d’Ivoire, for example, the NTD program manager attended regular high-level health ministry meetings on the pandemic. Notably, in Togo, the national representative of Act | West’s partner agency is a member of the national COVID-19 task force and has contributed to discussions and decision-making on the pandemic.

The case studies, developed between April and July 2020, led to a list of key readiness indicators for assessing the readiness of countries to resume interventions:

1. The availability of face masks and other necessary personal protective equipment for healthcare workers, community drug distributors, supervisors, and others directly involved in NTD activity implementation and for community members involved in facilitating the MDA.
2. A risk assessment conducted and NTD-specific risk communication and community engagement strategy developed.
3. The availability of standard operating procedures for NTD activity implementation in the context of COVID-19, including mitigation measures such as use of face masks, physical distancing of at least two meters between individuals, hand hygiene measures, and other steps relevant to the specific activity.
4. The operational readiness of the national NTD program to conduct the activities effectively, including the availability of funds (e.g., contract approvals, sufficient funds to cover additional costs, such as face masks or larger conference rooms to ensure physical distancing), drugs and/or diagnostic tools, and human resources.
5. Authorization from the national authorities to restart NTD activities, as well as their position on conducting other community-based public health interventions. This also included lifting of travel restrictions within the country, where necessary for the activity to move forward.

### Development of tools to monitor the resumption of NTD activities.

The Act | West team developed three key tools and/or processes to monitor activities as countries resumed: a supervisory checklist, a personal protective equipment tracker, and in-process activity monitoring and reporting process.

#### The COVID-19 NTD supervision checklist.

Act | West initiated the development of a COVID-19 NTD supervision checklist to guide the implementation of standard operating procedures to limit SARS-CoV-2 transmission during activities and provide corrective measures during field activities (Supplementary Material 2). The checklist included questions on field preparation, monitoring of crowd control, logistics, face mask availability and use, availability of appropriate training space, and whether country COVID-19 mitigation measures were followed. The checklist was adapted to an electronic data capture format and used in Guinea, Senegal, Benin, Cameroon, Burkina Faso, Niger, Mali, and Sierra Leone. The introduction of electronic data capture for MDA supervision was new in the Act | West program, and it proved to be an effective and useful tool for real-time data collection, management, analysis, and sharing. From the national program side, the checklist was used to identify areas to improve adherence to their procedures; from the partner side, the tool was used to identify whether additional support may be needed to follow mitigation measures.

#### The personal protective equipment tracker.

Because national COVID-19 guidelines vary by country, an important aspect of the tracker was to assess the existence of policies, such as mandatory wearing of face masks in public places, as NTD activities restarted. Second, this tracker collated data related to face mask availability for activities. It enabled the national NTD programs, Act | West program implementing partners, and donors to assess funding sources for their procurement, stockpiles, costs in local markets, and the possibility of procurement in remote areas.

#### In-process activity monitoring and reporting process.

In collaboration with USAID, the Act | West program developed and implemented field monitoring tools to ensure national mitigation measures were able to be implemented and to support field teams to take corrective measures where needed. To verify on-the-ground realities, the monitoring system included the following parameters, which were reviewed with USAID every other week as activities restarted in the supported countries:

• Access to and use of personal protective equipment, especially face masks, by survey participants and field workers
• Transportation and logistics involved in resuming activities
• Ability of supervisors to ensure physical distancing and control crowds
• Community perceptions of the continuation of NTD control activities
• Ability to follow mitigation measures while implementing NTD activities and addressing any difficulties in adhering to them
• Photos and videos were taken in the field and incorporated into regular reporting to the donor.

## RESULTS AND DISCUSSIONS

### Key findings.

In all 11 Act | West–supported countries, the national NTD programs were able to successfully restart activities between August and November 2020. By the end of November 2020, eight countries had implemented MDA for one or more diseases, and nine countries had conducted assessments for different diseases (mainly for lymphatic filariasis and onchocerciasis) (Table 1). More than six million persons across 289 districts were treated for one or more diseases, and 156 districts were surveyed. Despite the large number of communities and individuals that participated in these activities, in the two-week period after each of the field activities, national COVID-19 task forces did not report any surges in newly reported COVID-19 cases in the districts where NTD activities had resumed. However, we note this with caution, as we acknowledge that this may be due to lack of information about, or access to, testing sites for COVID-19 or limitations to surveillance. Also, there was no noticeable drop-off or turnover in community drug distributors. Overall, communities were enthusiastic about re-starting NTD activities, and no widespread public hesitancy was observed through the methods and tools used.

**Table 1:** Surveys and MDA implemented or ongoing between August and November 2020 with Act | West support

Country	Type of Survey	No. of districts where surveys were conducted/planned	Disease(s) targeted for MDA	No. of districts where MDA was conducted/ planned
Benin	TAS 2	9/9	Onchocerciasis/lymphatic filariasis	0/48
	Pre-re-TAS	4/4	Schistosomiasis/soil-transmitted helminths	0/42
Burkina Faso	Pre-TAS	3/3	Lymphatic filariasis	3/3
	TAS 1	1/1	Onchocerciasis/lymphatic filariasis	2/2
	TAS 2	2/2	Onchocerciasis	2/2
			Schistosomiasis	3/33
Cameroon	TAS 2	36/36	Onchocerciasis	77/113
			Trachoma	0/2
Côte d'Ivoire	Pre-TAS	46/46	Onchocerciasis/lymphatic filariasis /soil-transmitted helminths	74/75
			Trachoma	11/11
Ghana	Re-pre-TAS	5/5	Onchocerciasis/lymphatic filariasis	0/12
			Onchocerciasis	0/124
	TAS 1	0/3	Schistosomiasis/soil-transmitted helminths	0/89
Guinea	N/A	N/A	Onchocerciasis/lymphatic filariasis/schistosomiasis/ soil-transmitted helminths	19/19
			Trachoma	1/1
Mali	TAS 1	15/15	Schistosomiasis/soil-transmitted helminths	10/35
	TAS 3	7/14		
Niger	Pre-re-TAS	2/11	Trachoma	4/4
Senegal	Pre-TAS	14/14	N/A	N/A
Sierra Leone	N/A	N/A	Onchocerciasis/schistosomiasis/soil-transmitted helminths	0/149
			Schistosomiasis	9/9
Togo	Onchocerciasis Stop-MDA surveys (Epidemiological)	12/12	Onchocerciasis	35/36
			Schistosomiasis/soil-transmitted helminths	39/39

Of note, discussions among national programs, partners, and donors in the trachoma community resulted in a felt need to further assess personal protective equipment needs beyond face masks during trachoma surveys. During these surveys, a trained ophthalmic technician examines the eyelids of survey participants up close with a magnifying ocular loupe. During the examination, the examiner’s face is just inches from that of survey participants. Although the examination may take only about a minute per person, up to several thousand individuals may be examined per evaluation unit; therefore, it was felt that there was the potential for a higher risk of SARS-CoV-2 transmission during trachoma surveys than many other types of community-based NTD activities. Consequently, trachoma surveys did not resume with Act | West support during this timeframe.

Noticeably, the procurement of face masks and access to globally donated products were a challenge. There was significant variation in cost and available stockpiles across countries (Table 2). The national NTD programs managed to resolve the gaps in face mask provision a few months after the pandemic’s onset using various resources, including government funds as well as external funding from partners and other stakeholders.

**Table 2:** Country face mask (FM) data for NTD activities as of August 12, 2020

Country	Is wearing FMs mandatory in public places? (yes/no)	Are FMs available, produced or procured locally? (yes/no)	Unit cost estimates for FMs (USD)	Existing FMs in country stockpile (n)	Source(s) of FMs and dates received	Estimated need for FM (n)	Unmet need as of 8/12/2020 (n)	Source(s) (grants/donations from partners, donors, or government)
Benin	Yes	Yes	$0.37	0	N/A	14,129	14,129	Act | West
Burkina Faso*	Yes	Yes (cloth masks)	Cloth mask: $0.60 Medical mask: $0.90		Unknown	94,083	1,200	World Bank Burkina Faso State Other technical and financial partners.
Cameroon*	Yes	Yes	$1.00 for cloth masks	0	N/A	84,018	80,168	Act | West MoH to cover
Ghana	Yes	Yes	$1.11 per mask	0	0	122,699	122,699	NGOs
Cote d’Ivoire*	Yes	Yes	$0.43	NK	N/A	64,392	30,000	MoH/Cote d’Ivoire
Guinea	Yes	Yes	Cloth masks: $0.50	Local vendor can provide 5 million/ week. Stockpile NK.	Local	122,022	122,022	Donation from donors
Mali	Yes	Yes	$0.88 per cloth mask.	0 for NTD activities	N/A	39,702 for SCH MDA) 3,468 for TAS	43,170	USAID/Mali World Bank Project Sightsavers
Niger	Yes	Yes	$ 0.17 for cloth masks	NK	ONPPC	180 000	160 000	Government, MoH, donors
Senegal*	Yes	Yes	$0.71	0	N/A	155 392	155 392	Act| West
Sierra Leone	Yes	Yes	$0.51 for cloth masks	NK	NK	FY20 73,910 FY21 76,028 Total: 149,938	FY20 73,910 FY21 76,028 Total: 149,938	USAID Act | West
Togo	Yes	Yes	$0.14 for a box of 72	NK	NK	3,700	0	Government of Togo

MoH = Ministry of Health; N/A = not available; NK = not known; ONPPC = Office National des Produits Pharmaceutiques et Chimiques/National Office for Pharmaceutical and Chemical Products; FY = fiscal year.

* Countries receiving COVID-19 assistance from the U.S. government that may include purchase of FMs.

The countries developed strategies to disseminate messages to prevent the spread of SARS-CoV-2 and facilitate social mobilization during NTD activities. In Côte d’Ivoire, for example, the national program carried out training sessions for journalists and reporters to preempt rumors and mitigate their potential to hamper community mobilization during NTD campaigns. The training focused on responding to “fake news” and provided clear and concise information about the NTD program and its activities. Subsequently, the survey teams successfully reached the required sample sizes during surveys and achieved sufficient treatment coverage during the lymphatic filariasis and onchocerciasis MDA.

### Best practices, innovations, and lessons learned during MDA and disease-specific assessments.

*Best practices and lessons learned*. The national programs in Guinea, Benin, and Cameroon implemented systematic SARS-CoV-2 testing of supervisors and NTD program staff before leaving the capital city for activities in other districts as part of national COVID-19 prevention guidelines. Testing was coordinated between the national NTD program and national COVID-19 task force. At least two persons were excluded from participation in field activities after a positive COVID-19 test. During surveys, survey teams also conducted on-site temperature checks using laser thermometers for health workers and activity participants.

For NTD surveys, registration was conducted house-to-house, and selected participants were invited to present at the survey site in a specific order (by name or number) or in small groups of two to three individuals in Mali (TAS) and Togo (stop onchocerciasis MDA survey). In Mali, staff members who traveled to the field participated in an orientation to review COVID-19 mitigation measures, including requirements for activity resumption and donor requirements. Participants queuing generally respected physical distances of 2 meters, with queue management by health personnel supporting the survey team. During the pre-TAS in Côte d’Ivoire, due to the extra time required to ensure proper rollout of measures to prevent transmission of SARS-CoV-2 in the field, people initially waited while standing in long queues for extended time periods to have their fingers pricked for antigen testing. In collaboration with the local communities, the survey teams implemented crowd management approaches by requesting that the communities provide chairs. They then spaced the chairs two meters apart to ensure proper physical distancing and resolve the discomfort of standing for a long time. Across all sites, as part of the risk communication and community engagement strategy, it was important for the community members to understand that fingerstick blood sampling was not related to COVID-19 testing or vaccine trials.

For training, most national programs found spacious, well-ventilated rooms or used outdoor venues. Most preparatory meetings and surveys were performed outdoors. Outdoor training for MDA drug distributors was also initiated in some districts in Sierra Leone. The Act | West program made financial provisions to rent additional rooms where needed. Handwashing stations with soap were installed in front of the training rooms, and physical distancing was observed during training, lunch, and coffee breaks.

In all countries, NTD program staff, other health workers, and community drug distributors were required to wear face masks during all activities. Face masks were additionally distributed to survey participants in in Benin, Ghana, and Côte d’Ivoire. Before arriving at the station where participants had their fingers pricked for antigen testing during surveys for lymphatic filariasis, participants washed their hands with soap and then proceeded to the testing station while observing physical distancing and properly wearing face masks covering their noses and mouths. Wherever feasible, if survey respondents arrived without a mask, they were asked to return home for their own masks. At the station, the laboratory technician instructed the participant to sit sideways, facing away from the technician, to minimize face-to-face interactions.

For MDAs, as during pre-pandemic MDAs, most treatments were distributed door-to-door rather than at a fixed location, where possible, to minimize public gatherings (unless MDA was school-based). Although school-based distribution is operationally convenient and effective in reaching high coverage for school-age children, they are fixed-point mass gatherings and more prone to the spread of SARS-CoV-2 infection. Drug distributors used spoons and trays to reduce contact during drug administration. Handwashing was promoted by the drug distributors and MDA supervisors and implemented by household members during home visits for drug distribution. In Guinea, drug distributors used a long stick to handle the dose pole, which allowed them to maintain several feet of distance between themselves and the participants whose height they needed to measure to ascertain the correct dosage of NTD medicines. In most countries, height was measured by asking the participant not to face the drug distributor or the volunteer taking the measurements. Other contactless methods of measuring height were effectively implemented in Niger, Côte d’Ivoire, and Guinea during MDA by using dose poles drawn on or propped against walls in classrooms or houses.

#### Capitalizing on local initiatives.

The national programs used local initiatives and approaches to reach out to the targeted populations and achieve successful results while ensuring compliance with measures to limit SARS-CoV-2 transmission. The national program in Sierra Leone employed local tailors to produce washable cloth masks and made them available to schoolchildren and teachers throughout the country. The initiative was successful and allowed continuation of NTD activities, especially school-based deworming.

In Benin, where moto-taxis (called *zemidjans*) are the quintessential mode of transportation, the national program used *zemidjan* drivers to reach out to several groups of people, including traders, peddlers, and market women. The market women were particularly instrumental in further spreading sensitization messages on COVID-19 prevention in markets and surrounding areas and amplifying the mobilization for MDA.

The pandemic has caused some community members to be fearful or hesitant to take NTD drugs, as well as participate in surveys; however, this was only observed in a few districts. To respond, national programs deployed strategies to minimize refusals by working with influential and well-respected community leaders. In Cameroon, in the first few days of the TAS field data collection in some urban communities, some community members did not initially want to have their children tested because they feared that the survey teams had been sent to infect their children with SARS-CoV-2. The teams then solicited the support of traditional and religious authorities, who led community sensitization activities. Subsequently, the teams were able to enroll enough people to cover all the targeted geographic clusters.

### Assessing the readiness to restart.

At the beginning of the pandemic, the national authorities in Benin, Burkina Faso, Guinea, Côte d’Ivoire, and Niger imposed sanitary belts around capital cities, the areas most affected by transmission of SARS-CoV-2, to restrict travel in and out of urban settings and limit disease spread. Because central-level NTD staff needed to travel across the country to support the field interventions, the national programs waited until the restrictions were lifted to resume program activities. In the case of Guinea, where restrictions were still in place, central-level MDA campaign staff were tested for SARS-CoV-2 before travel to the field. In all supported countries, Act | West worked with the national NTD programs to assess whether they felt ready to conduct activities. As part of these discussions, the predetermined key readiness indicators were reviewed to determine whether they had been met. If not, discussions on challenges to do so were held. For example, the closing of schools impeded the restart of deworming in some countries, such as Senegal and Ghana.

### Additional financial resources, time, and staff.

Preparing for restarting activities, including coordinating the logistics necessary, required extra time and resources. An estimated two to three additional hours per site were necessary for pre-TAS in Senegal, and an additional day was needed at each pre-TAS site in Côte d’Ivoire. Also in Côte d’Ivoire, the national NTD program engaged additional volunteers/community drug distributors to assist with crowd control. In Cameroon, during previous TAS surveys, the national program would work with one community mobilizer per cluster. In the context of the pandemic, the national program hired three community mobilizers per cluster to ensure physical distancing and manage crowd control. Each mobilizer helped ensure order, avoid congestion at the site, and manage traffic flow. A maximum of 10 children was taken to the survey testing area at a time, after receiving consent from their parents. The community mobilizers also assisted the heads of survey teams by disseminating messages about COVID-19 prevention in communities.

Several countries experienced cost increases related to mitigation measures, logistics, and field implementation. An internal, unpublished rapid assessment in Benin, Togo, Côte d’Ivoire, and Guinea between July to September 2020 found a significant variation of costs depending on the type of field activity and country. Between $3,000 to $26,000 in additional costs were incurred to restart NTD activities, compared with the usual costs incurred under nonpandemic circumstances. This variation was dependent on the type of NTD activity and the country in which the activity took place. In general, the key cost drivers were the following:

1. Transportation—the need to rent more vehicles to respect physical distancing (maximum of three persons per car) where feasible;
2. Training—when trainings were not held outdoors, the requirement for extra and/or more spacious training rooms to accommodate physical distancing;
3. Per diem—due to the longer timeframes and in some cases, increased personnel, needed to conduct activities; and,
4. Hygiene—handwashing stations, hand sanitizer, soap, contactless laser thermometers, face masks, and COVID-19 testing of NTD program staff where it was local policy and feasible.

### Next steps.

In the absence of guidelines or standard operating procedures specific to trachoma surveys, planned trachoma impact surveys and surveillance surveys were put on hold across the Act | West, program as described earlier. Resumption of these surveys is likely as soon as countries develop trachoma-specific mitigation procedures based on ongoing operations research into the usage of special combined face shields and ocular loupes.

We did not collect robust costing data on additional funds needed for field operations, training rooms, crowd control, transportation, and extra staff. In the future, the national programs and their stakeholders should design and implement cost studies to capture the real costs of restarting NTD activities and the financial impact of the COVID-19 pandemic.

The Act | West team implemented rigorous monitoring and reporting systems, including in-process supportive supervision, to address challenges encountered during field implementation. The monitoring system included field photography and video for review and for implementing corrective measures as needed. The best practices and innovations that were successfully implemented during NTD field activities in the context of the pandemic should be shared with NTD program managers and recommended to countries resuming NTD activities.

## Supplemental Material


Supplemental materials
