# Supplementary material for: Restarting Neglected Tropical Diseases Programs in West Africa during the COVID-19 Pandemic: Lessons Learned and Best Practices
Source: Am J Trop Med Hyg. 2021 Oct 20;105(6):1476–82. doi: 10.4269/ajtmh.21-0408 (PMC8641318; doi:10.4269/ajtmh.21-0408)
Supplement: Supplementary file 1 [file tpmd210408.SD1.pdf]

Considerations for Resuming NTD programs ("NTD Restart Matrix")

Country:
Date:
Name and title of the person filling the form:

	Domain	Details	Comments	Source of information
1	**RISKS**			
	COVID-19 Situation (as of DATE)			
	No. of Cases Reported			
	Trends/No. of incident cases for the last 14 days			
	Projection of the number of cases for the next 7, 14 and 21 days (if known)			
	Contact tracing			
	No. of Deaths Reported			
	Location of Cases/Deaths (e.g. capital city, limited to certain regions, etc.)			
	Status of COVID-19 testing/availability			
	Other important considerations (e.g. doubling rate, etc.)			
	Epidemiological situation of the NTDs AND COVID-19 in areas targeted to restart MDA?			
	Epidemiological situation of the NTDs AND COVID-19 in areas targeted to restart DSA?			
	**Risk Assessment & Mitigation Plan**			
	**Risk Assessments**			
	Provide details for any risk assessment(s) undertaken for NTD activities			
	Provide details for any NTD Risk Assessment tools piloted in the country.			
	**Mitigation Plans**			
	Detail any plans to conduct non-health related field activities (example – income generating activities for groups of women) in areas with no COVID-19 /programming based on prevalence.			
	Describe the risk mitigation plan in place for community health activities at-large -e. g NTD MDAs, bed nets distribution (if applicable).			
	Support/assistance from implementing partners (IPs)			
	Describe the risk mitigation plan specifically for campaign and/or NTD activities (e.g. modified/adapted MDA/DSA strategies to minimize direct contact/exposure such as			

	remote/virtual trainings, small groups trainings, door-to-door versus fixed point distribution etc.).			
	Do more training sessions need to be held in multiple or larger venues utilized to ensure physical distancing?			
	Do training curricula need to be modified/time added to training to incorporate COVID-19 prevention aspects?			
	Does the MDA timeline need to be extended to accommodate COVID prevention measures?			
	Are there any discussions underway, or plans to combine/integrate interventions (community or other) to reduce exposure?			
	What adaptive measures are implemented to avoid transmission of COVID 19 during school-based distribution?<br><br>For example: Could the dose-poles be drawn on a wall in the classroom to minimize contacts/exposure during measurements for drug distribution?			
	Does the government plan to widely distribute guidance or policies around WASH? Would NTDs be part of it?			
	Could adaptive measures potentially introduce selection biases, discrimination/segregation of groups of people regarding MDA?			
	Please comment about any additional time and resources needed to plan and conduct an MDA or a DSA given the COVID situation			
	**Communication about COVID-19 and Social Mobilization for NTDs**			
	Is a specific COVID-19/NTD risk communication and community engagement plan/strategy developed?			
	Are NTD communication plans being adjusted to include COVID-19 education and messaging?			
	Are supervisors, teachers, CDDs and health workers fully aware and involved in the communication about NTD campaigns/interventions in the context of COVID 19			
	Are local NTD implementing partners involved and supportive of the communication efforts about NTDs in the context of COVID 19?			

	Are local media, community radios, TV, social media and all relevant channels used CONSISTENTLY for messaging and education about COVID 19			
**2**	**POLICIES**			
	**National Government**			
	Briefly explain the structure, membership, and function of the National COVID-19 Response Task Force or Coordination Unit (or equivalent)			
	Does the national COVID-19 response unit has decentralized entities in the regions and districts?			
	Status of key public measures by national authorities (e.g. limitation of the number of people allowed in a meeting, gathering, travel authorizations etc.)			
	Do the appropriate authorities above the MOH approve of starting NTD activities? If so, how is this documented officially?			
	Does the highest authority (above MoH) in the country approves the restarting of NTD activities?			
	**Ministry of Health**			
	Does the MOH approve of starting NTD activities?			
	Is the NTDP and MOH aligned with the decision to resume NTD activities?			
	Are central level staff being tested for COVID-19 before being allowed to undertake field activities?			
	**USAID Washington (to complete)**			
	Has USAID/W issued any COVID-19 guidance to IPs operating in country?			
	Has the USAID/Mission issued any COVID-19 restrictions that would apply to implementation of NTD field activities?			
	**WHO Country Office**			
	What is the WHO country office position on resuming NTD field activities?			
	How has the WHO Country office been engaged in risk assessment or mitigation for NTD activities?			
	Does the WHO country office attend regular COVID-19 meetings organized by the MoH?			

	**Schools**			
	Are schools open? Or is there a date for re-opening?			
	What are the policies in place for reducing transmission in schools?			
	Does each school have a handwashing facility? (tap water, dipping water in a station?			
	Does the school system have a reporting system for COVID-19?			
	**Status of other Community-based Interventions (non-NTD)**			
	Provide details for any other public health mass campaigns underway (e.g malaria, vitamin A, immunization etc.).			
	Are there any activities proceeding that MDA or DSAs could be feasibly linked to?			
**3**	**CAPACITY AND READINESS**			
	**Drug Supply**			
	Are drugs available and in the right place?			
	Are any NTD drugs expiring in the next quarter as a result of COVID-19 delays?			
	Are all necessary commodities available (e.g. tests for DSAs, etc.)			
	**PPE Status**			
	What is the availability of PPE (hydro-alcoholic solutions, facemasks, gloves etc.) to the NTD Program staff and community drug distributors			
	Is PPE procurement available on the local market?			
	If PPE procurement is needed, who will procure it?			
	**Human Resource Availability**			
	Are NTDP staff availability for NTD activities (e.g. supervision of field activities)?			
	Are key Regional/District Level staff available for NTD activities (e.g. supervision)?			
	Are CDDs available for training/drug distribution and willing to participate?			
	Have NTD staff at any level been re-assigned to COVID-19 activities?			
