# Supplementary material for: Restarting Neglected Tropical Diseases Programs in West Africa during the COVID-19 Pandemic: Lessons Learned and Best Practices
Source: Am J Trop Med Hyg. 2021 Oct 20;105(6):1476–82. doi: 10.4269/ajtmh.21-0408 (PMC8641318; doi:10.4269/ajtmh.21-0408)
Supplement: Supplementary file 2 [file tpmd210408.SD2.pdf]

USAID DU PEUPLE AMERICAIN · Act to End NTDs WEST · fhi360 THE SCIENCE OF IMPROVING LIVES

COVID-19 / NTD DSA SUPERVISION CHECKLIST

<u>Instructions:</u> *The supervisor should complete the sections for which she or he has information or obtain it by direct field observation, consulting documents on-site, or interviewing field team members or key informants. supervisors should select "N/A" if the item doesn't apply to the local context.*

SUPERVISION CHECKLIST FOR NTD DISEASE-SPECIFIC SURVEYS	
**Supervisor (first and last name):**<br>**Date:**	- District:<br>- Subdistrict:<br>- Village/School:
Phone number	
Type of supervised activity? Please circle one:<br>Pre-TAS - TAS - TIS -TSS - Pre-STOP OV MDA - SCH/STH survey	
**Number of new and active COVID -19 cases in the district (or site) in the last 2 weeks:**	

	Please check one of the boxes below			
**I - Preparation and logistics**	**Yes**	**No**	**N/A**	If no, are corrections brought up Yes/No?
Supervisors are tested for COVID-19 before travelling to the field				
Are materials, equipment, tools pre-arranged and deployed on the site to minimize movements and disturbance by the survey team?				
Are there enough masks, gloves, hand sanitizer available in the field?				
Are there enough face-shields available in the field?				
The means of transportation to and from the site are enough for the staff to respect a minimum of social distancing in each vehicle (maximum four persons per vehicle including the driver)				
**II – Planning**				
COVID-19 mitigation measures have been communicated and related documents are made available to local authorities and medical staff				
The exact date, time and duration of field operations are known to the target communities a week before the launch				
No other parallel (or concomitant) activities -capable of disturbing or confusing the survey -will occur during NTD activities (Example: Vaccination campaign, nutrition/vitamin A, mass protests, markets, recreational activities, traditional etc.) in the site				
**III - Training**				
The training room is well ventilated, free of any clustering of people and has good air circulation between the rows				
Have participants been checked for symptoms (temperature, or clinical				

	symptoms) and are proper procedures followed?				
	Furniture and equipment are properly desinfected before and after the sessions				
	There are fewer than 50 persons in each training room				
	The physical distance of at least 2 meters between participants is always observed in the room				
	Everyone in the room wears a mask properly (covering mouth and nose)				
	Is a handwashing device or hand sanitizer available in the room				
	All the people in the room washed their hands with soap or used hand sanitizer before and after the training				
	The phone numbers and addresses of all participants were recorded (to allow tracing and tracking later -on if needed)				
**IV - Communication and Social Mobilization**					
	Messaging on COVID-19 and NTDs was communicated to everyone at the site and included COVID-19 prevention measures				
	IEC material on COVID-19 is available in the field				
	The social distancing of 2 metres between participants is enforced during messaging and social mobilization (through local communication channels)				
**V - Mitigation measures during field survey**					
	Are crowd control measures in place?				
	The social distancing of 2 meters between participants is enforced during any registration or pre-selection of participants				
	Are most people wearing masks in the community where the survey is taking place				
	Are the participants selected for the survey wearing masks?				
	Do all the officers involved in the survey wear a mask?				
	Is the mask properly worn by participants, community volunteers and investigators?				
	Are survey participants coming per selected household or pre-assigned groups to the site				
	The social distancing of 2 metres between participants and all other field officers is observed during the survey				
	The social distancing of 2 meters between participants is strictly applied during coffee breaks and lunch				
	The social distancing of 2 meters between participants is respected during photos (if any photo is taken)				

	The temperature of each participant is taken at a distance with a laser thermometer				
	The temperature of each surveyor/operator has been checked at a distance with a laser thermometer				
	All the technicians involved in blood collection and sample handling are wearing masks and gloves				
	Availability of Alcohol-based hand sanitizers on site				
	self-monitoring of COVID-19 symptoms is done by DSA actors (Supervisors, Investigators and aides on a daily basis				
	Availability of on-site functional handwashing devices with soap and water				
	Participants in the survey use hand sanitizer or wash their hands before being examined/finger pricked for blood sampling				
	Operators/surveyor and technicians use hand sanitizer before and after each participant's examination				
	The equipment and furniture used are regularly disinfected and cleaned with a solution of disinfectant in the field				
	People exhibiting COVID-19 symptoms (e.g. coughing, exhaustion, difficulty breathing, sneezing etc.) will not participate in the survey and referred to the nearest health center				
	Are biomedical waste management devices (trash can or bin with lid) in place at the site?				
	**VI. Community Perception**				
	Are rumors in the community reported to the supervisors by aides or community leaders				
	Local people recognize the COVID-19 pandemic as a reality in the country				
	Parents of students are sensitized and cooperate with the team of investigators				
	Local authorities (traditional, administrative and religious leaders) fully adhere to mitigation measures				
	Participants and local communities have not expressed any concern or issues before and during field operations				
	The local population is satisfied with the hygiene and protective measures put in place before and during the field activity				
	**V- Problems/Issues encountered**				

Supervisor's signature

Supervision Checklist - COVID-19 Barrier Measures during MDA

Region:
District:
Health center:
Supervisor team:
Date:

Instructions: Please consider your country SOP when completing this checklist and select "N/A" if the measure does not apply to your context. The first section (COVID-19 epidemic) is a questionnaire for health district staff involved in NTD activities. The remaining sections are observations, feelings and actions of the supervisor filling in this checklist. If a teacher is distributing the drugs in a school-based distribution, consider them as the "CDD".

Questions	Please check one of the boxes below			
	**Yes**	**No**	**N/A**	If no, are corrections brought up Yes/No?
**1. COVID-19 epidemic:**				
Are there any known positive cases in the district in the last two weeks?				
If yes, what is the number of cases?				
Have supervisors been tested for COVID-19 in the week before travelling for supervision?				
If yes, did anyone test positive?				
If yes, NTDP procedure followed?				
Is self-monitoring of COVID-19 symptoms done by MDA actors (Supervisors and CDDs) on a daily basis?				
If yes, is self-monitoring reported to chief of mission?				
Were COVID-19 preventive and barrier measures (social mobilization, hand washing, physical distancing etc.) covered during the training of CDDs?				
If yes, were these messages included in communications via public criers, CDDs, radio?				
**2. Hygiene measures**				
Are participants washing their hands or using hand sanitizer before receiving treatment?				

USAID FROM THE AMERICAN PEOPLE | Act to End NTDs WEST | fhi360 THE SCIENCE OF IMPROVING LIVES

	Are CDDs wearing gloves during MDA?				
	Are CDDs using clean tools (e.g. spoons, trays, bottle caps) to give pills to participants?				
	If no, are CDDs touching pills or participants with their hands?				
**3. Barrier measures during MDA**					
	Are the CDDs wearing a mask/facial covering during activities?				
	If yes, is the mask/facial covering being worn properly (I.e. covers nose and mouth)?				
	Are the participants wearing a mask/facial covering during the MDA?				
	Is the physical distancing (2 meters) being respected between CDDs and participants during MDA including inside a house, if applicable? (Except for when the CDD physically gives the pills to the participant).				
	Are participants touching the dose pole during their measurement (with any part of their body)?				
	If yes, is the dose pole washed or disinfected after each household?				
	If no, how is the dose pole being held?				
**4. MDA strategy** (please ignore the questions related to the MDA strategy that you are not using currently in your context if using paper format for the survey)					
☐ Door to door<br>☐ Fixed point<br>☐ School based					
**Door-to-Door**					
	Only household members being treated together (without other people from another household)?				
	If no, **which** specific prevention/barrier measures are being taken?				
	Are community members following CDDs from house to house				
	If yes, what crowd control measures are in place				

	Are CDDs washing their hand or using hand sanitizer before entry and after exit of house compound?				
	**Fixed Point**				
	Are people from the community crowding around CDDs during MDA (within 2 meters)?				
	If yes, what the crowd control measures in place				
	Are most people from the community wearing a mask outside their homes?				
	**School-based**				
	If school-based, are students being treated outside the classroom in a school-based MDA?				
	If school-based, are there hand washing stations in the school compound?				
	Are students wearing masks in the school compound?				
	Is physical distancing of 2 meters respected in the school compound?				
	**5. Impact of COVID 19 on MDA participation**				
	Were the CDDs generally reluctant to participate in the MDA due to COVID-19?				
	If yes, could you provide more information				
	Were the communities generally reluctant to participate in the MDA due to COVID-19?				
	If yes, could you provide more information				
	Did you take photos and or make short videos of the MDA activities in COVID-19 context and share with partners and donors?				
	if yes, were participants asked to tick a box at registration giving permission to use their photos/videos publicly (and told that they will still receive the drugs if they tick 'no')?				
	Any other thoughts or observations including logistics				

	Corrective measures put in place in case of insufficiencies observed ___________________________
